# Understory vegetation diversity, soil properties and microbial community response to different thinning intensities in *Cryptomeria japonica* var. *sinensis* plantations

**DOI:** 10.3389/fmicb.2023.1117384

**Published:** 2023-02-28

**Authors:** Kai-Li Liu, Bo-Yao Chen, Bin Zhang, Rui-Hui Wang, Chun-Sheng Wang

**Affiliations:** ^1^College of Forestry, Central South University of Forestry & Technology, Changsha, China; ^2^Research Institute of Tropical Forestry, Chinese Academy of Forestry, Guangzhou, China; ^3^National Long-term Scientific Research Base of Central and Subtropical Forestry, Changsha, China

**Keywords:** soil microbial community composition, microbial function, thinning, soil properties, understory vegetation

## Abstract

**Introduction:**

Soil microorganisms are the key factors in elucidating the effects of thinning on tree growth performance, but the effects of vegetation and soil on the species composition and function of soil microorganisms after thinning are still not well elaborated.

**Methods:**

The effects of thinning on understory vegetation diversity, soil physicochemical properties and soil microbial community composition were investigated in a thinning trial plantation of *Cryptomeria japonica* var. *sinensis*, including four thinning intensities (control: 0%, LIT: 20%, MIT: 30% and HIT: 40%), and the relationships of the microbial community structure with the understory vegetation diversity and soil properties were assessed.

**Results:**

The results showed that thinning had a greater effect on the diversity of the shrub layer than the herb layer. The soil bulk density and the contents of soil organic matter, total potassium and nitrogen increased with increasing thinning intensities. The Shannon and Chao indices of soil bacteria and fungi were significantly lower in the LIT, MIT and HIT treatments than in the control. Thinning can significantly increase the abundance of *Proteobacteria* and *Actinobacteria*, and higher thinning intensities led to a higher relative abundance of *Ascomycota* and a lower relative abundance of *Basidiomycota*, *Rozellomycota*, and *Mortierellomycota*. Redundancy analysis indicated that soil physicochemical properties rather than understory vegetation diversity were the main drivers of microbial communities, and fungi were more sensitive to soil properties than bacteria. Functional prediction showed that thinning significantly reduced the potential risk of human diseases and plant pathogens, and the nitrogen fixation capacity of bacteria was the highest in the HIT treatment. Thinning significantly increased the relative abundance of cellulolysis and soil saprotrophs in bacteria and fungi.

**Conclusion:**

The findings provide important insights into the effects of thinning on *C. japonica* var. *sinensis* plantation ecosystems, which is essential for developing thinning strategies to promote their ecological and economic benefits.

## 1. Introduction

Soil microorganisms play a crucial role in nutrient decomposition, absorption, and transformation in terrestrial ecosystems (Soong et al., 2019; Tian et al., 2019; Averill et al., 2021). The diversity and abundance of soil bacteria and fungi not only affect nutrient mineralization and cycling but are also sensitive to ecological environmental conditions, such as soil properties (Stark et al., 2012; Shen et al., 2013; Urbanová et al., 2015; Lladó et al., 2018), vegetation diversity (Liu et al., 2021; Shang et al., 2021), and climatic conditions (Bona et al., 2021). Soil properties are considered crucial factors influencing soil microbial activity and community structure, which have become a research hotspot in the field of the soil microbiome. For example, pH is frequently considered the main driver of microbial community structure in soil (Lammel et al., 2018; Ni et al., 2021; Queiroz et al., 2021); soil moisture strongly affects the fate of soil nutrient diffusion (Lei et al., 2022), and is also an important factor influencing the soil microbial community (Goransson et al., 2013; Lei et al., 2021); and the composition and diversity index of bacteria are significantly correlated with the contents of total nitrogen, available nitrogen and available phosphorus in soil (Zhang et al., 2016). However, soil nutrients are one of the factors influencing vegetation growth. In the process of nutrient recycling, nutrients are taken up and utilized, partly retained in plants, and partly returned to the soil as litter; and then, nutrients are released by the decomposition of litter materials (Osman, 2013). Soil organic matters mainly come from litter, and their decomposition can be an important energy source for soil microorganisms (Yang et al., 2017; Phillips et al., 2019; Xu et al., 2020). Therefore, vegetation may also play an important role in changes in the soil microbial community.

As a traditional technique in forest management, thinning not only has direct effects on light transmittance and microclimate in forests (Son et al., 2021) but also indirectly affects understory vegetation diversity (Ares et al., 2009) and soil physicochemical properties (Wic Baena et al., 2013). Thinning practices influence the understory vegetation diversity, and affect the functions of the soil microorganisms (Dang et al., 2018). It has been proven that the diversity and community structure of aboveground plants and belowground microbial communities in forest ecosystems are closely correlated (Chen et al., 2015; Zhou et al., 2020). To date, the relationship between soil physicochemical characteristics and vegetation has been widely studied. However, research on the relationships of soil microorganisms with understory vegetation diversity and soil properties under thinning is still scarce.

*Cryptomeria japonica* var. *sinensis* is an excellent fast-growing timber species and is one of the main plantation species in the high-altitude subtropical areas of China (Zhang et al., 2021). In particular, the planting area of *C. japonica* var. *sinensis* has been nearly 200,000 hectares in the western parts of Sichuan Province, which is located in the upper reaches of the Yangtze River. These *C. japonica* var. *sinensis* plantations are important ecological shields of the Yangtze River, and play an important role in economic development and environmental protection in this region. Due to the high density of monoculture in the initial planting, low stand quality and ecological benefits occur in the *C. japonica* var. *sinensis* forest.

To improve stand quality and promote the ecological benefits of these plantations, we arranged a thinning trial in 2014 and investigated the understory vegetation diversity, soil physicochemical properties and microbial community compositions of the trial plantations 5 years after thinning. We hypothesized that thinning of *C. japonica* var. *sinensis* plantations would affect the soil microbial composition and diversity, which would be driven by the changes in understory vegetation diversity and soil properties. This study aimed to specifically address the following: (1) to understand the changes in understory vegetation diversity and soil properties after thinning at different intensities; (2) to explore the differences in soil microorganism composition and function under thinning at different intensities; and (3) to elucidate the relationships of soil bacteria and fungi with understory vegetation and soil physicochemical properties.

## 2. Materials and methods

### 2.1. Experimental site

The thinning trial *Cryptomeria japonica* var. *sinensis* plantation is located at Yangziling Forest Farm, Yaan City, Sichuan Province, China (29°47′37″N, 102°56′18″E). It belongs to the subtropical monsoon mountain climate with abundant rainfall. The annual mean air temperature, precipitation and humidity are 13.1°C, 1,800 mm and 79%, respectively. The plantation was established with a spacing of 2.0 m × 1.5 m in April 2006 with an area of approximately seven hectares. The soil is yellow loam with a pH value of 4.3–4.7. The mean altitude of the site is 1,539 m, and the slope is approximately 15°.

### 2.2. Experimental design

The thinning trial was carried out in October 2014, and arranged in a randomized complete block design with three replicates and four thinning intensity treatments, including no thinning (control), light-intensity thinning (LIT: 20% of the trees removed), moderate-intensity thinning (MIT: 30% of the trees removed) and strong-intensity thinning (HIT: 40% of the trees removed). Each plot was 600 m^2^ in size and was surrounded by buffer zones (5 m) to reduce potential edge effects. The stand density and height (m) and diameter at breast height (DBH) (cm) of each tree were measured for each plot just after thinning (October 2014) and October 2019. The tree growth performance of each treatment is presented in Table 1.

**Table 1:** Information on the thinning trial plantation.

Thinning intensity	Stand density (trees·ha^−1^)	Year 2014		Year 2019		Increase of each year	
		Mean DBH (cm)	Mean height (m)	Mean DBH (cm)	Mean height (m)	Mean DBH (cm)	Mean height (m)
Control	2,833 ± 25	11.50 ± 0.30b	6.80 ± 0.10d	14.10 ± 0.60b	11.02 ± 0.32d	0.53 ± 0.18c	0.88 ± 0.10a
LIT	2,266 ± 44	12.37 ± 1.75b	7.17 ± 0.15c	16.30 ± 1.25b	11.75 ± 0.46c	0.79 ± 0.05b	0.95 ± 0.03a
MIT	1,983 ± 85	13.27 ± 0.47ab	8.90 ± 0.30a	18.10 ± 0.66ab	13.37 ± 0.28a	1.09 ± 0.02a	0.90 ± 0.13a
HIT	1,700 ± 69	14.27 ± 0.23a	7.80 ± 0.10b	19.5 ± 0.46a	12.50 ± 0.56b	1.12 ± 0.13a	0.93 ± 0.07a

The values are the mean ± standard deviation (*n* = 3), followed by different letters showing significant differences in the four thinning treatments for each index at the 0.05 probability level.

### 2.3. Understory vegetation investigation and diversity analysis

Four 2 m × 2 m subplots in each plot and one 1 m × 1 m quadrat in each subplot were established to assess the diversity of the shrub and herb layers, respectively. In each subplot or quadrat, the number was counted, and coverage and frequency were measured for each understory plant species in October 2019. The Shannon index and Pielou index were calculated (Wang G. et al., 2021), and the relative species abundance was calculated to indicate the species richness for the shrub and herb layers.

### 2.4. Soil sampling and physicochemical analyses

In each plot, soil profiles were excavated routinely at five points, and soils were sampled with a cutting ring (100 cm^3^) at topsoil layers (0–15 cm) to measure soil bulk density (SBD). Two topsoil samples were then collected at each point. One was placed on ice in the field and was then transported promptly to the laboratory and stored at −80°C for DNA extraction, and the other was dried at room temperature for physicochemical analysis. For each purpose, soils from five points were mixed equally as one sample for each plot before further treatment or analysis.

SBD was measured by the cutting ring method (Grossman and Reinsch, 2002), and soil moisture (SM) was determined by oven drying soils at 105°C for 24 h. Soil pH was measured using a pH meter (soil to water ratio was 1:2.5) (Kenworthy et al., 1976). The total nitrogen content (TN) was measured by the Kjeldahl method using a 2,300 Kjeltec Analyzer Unit (FOSS, Sweden) (Bremner and Mulvaney, 1982). The contents of total phosphorus (TP) and total potassium (TK) were determined *via* the ascorbic acid colorimetric method and atomic absorption method, respectively. The soil organic matter content (SOM) was assessed using the dichromate wet combustion method and a visible spectrophotometer (Lefroy et al., 1993). The alkaline hydrolysis method, molybdenum blue colorimetric method (Tan et al., 2014), and a flame photometer (Lu, 2000) were used to measure the contents of available nitrogen (AN), available phosphorus (AP) and available potassium (AK), respectively.

### 2.5. DNA extraction, PCR amplification and sequencing

Soil DNA was extracted in triplicate from 0.25 g of each sample using an E.Z.N.A.® soil DNA Kit (Omega Bio-Tek, Norcross, GA, U.S.) following the manufacturer’s instructions. The extraction quality of DNA was detected by 1% agarose gel electrophoresis, and the concentration and purity of DNA were determined by a NanoDrop2000.

The V3-V4 region of the bacterial 16S RNA gene and partial fungal ITS regions were amplified by PCR using the primers 338F (5′- ACTCCTACGGGAGGCAGCAG-3′) and 806R (5′- GGA CTACHVGGGTWTCTAAT-3′), and ITS1F (5’-CTTGGTCATTTA GAGGAAGTAA-3′) and ITS2R (5’-GCTGCGTTCTTCATCG ATGC-3′), respectively (Manter and Vivanco, 2007). All amplifications were performed in 20 μl mixtures containing 4 μl of 5 × FastPfu Buffer, 2 μl of 2.5 mM dNTPs, 0.8 μl of each primer (5 μM), 0.4 μl of FastPfu Polymerase and 10 ng of template DNA. The amplification program included initial denaturation at 95°C for 3 min, followed by 27 cycles at 95°C for 30 s, 55°C for 30 s, and 72°C for 45 s, with a final extension at 72°C for 10 min. However, the PCR cycle number was 35.

The PCR products were purified using an AxyPrep DNA Gel Extraction Kit (Axygen Biosciences, Union City, CA, USA), and were quantified using QuantiFluor™-ST (Promega, USA) (Zhou et al., 2019). Subsequently, all the PCR products were pooled with equal molarity. Finally, the amplicons were pair-ended sequenced on an Illumina MiSeq platform (Illumina, San Diego, USA) by the Majorbio Bio-Pharm Technology Co. Ltd. (Shanghai, China).

### 2.6. Sequence splicing and annotation

The raw sequences were quality-filtered and merged using Fastp^1^ (version 0.19.6) and FLASH^2^ (version 1.2.11), respectively. Operational taxonomic unit (OTU) clustering and chimera removal were conducted using Upraise software^3^ (version 11) based on the 97% similarity threshold (Edgar, 2013). The taxonomic assignments of 16S rRNA and ITS sequence reads were determined using the bacterial SILVA reference database (Release138^4^) and the Unite reference database (Release 8.0^5^), respectively. For both databases, the Ribosomal Database Project (RDP) Classifier^6^ (version 2.11) was used to perform taxonomic annotation for OTU representative sequences, and the confidence threshold was set to 0.7 to obtain taxonomic annotation results. The sample sequence was flattened according to the minimum number to obtain standardized data for calculation of the Shannon index and Chao index according to Cao et al. (2022), and compositions of the soil microbial community were analyzed at the phylum level.

### 2.7. Determination of the metabolic and functional prediction

The functional annotation and prediction of metabolic or other putative ecological functions were assessed based on the Tax4Fun-KEGG (Wang et al., 2020) and FAPROTAX databases (Lu et al., 2022) for bacteria. Tax4Fun converts the SILVA-based OTU counts into functional or metabolic profiles and computed metabolic reference profiles based on the Kyoto Encyclopedia of Genes and Genomes (KEGG) database. The FUNGuild database (Nguyen et al., 2016) was adopted to predict the ecological and biological functions of ITS genes for fungi, which is a flat database hosted by GitHub.^7^

### 2.8. Statistical analysis

One-way analysis of variance (ANOVA) and a least significant difference multiple range test (*p* < 0.05) were performed to assess the significance that thinning affected the assayed soil physicochemical properties (SM, SBD, pH, SOM, TK, TP, TN, AN, AK and AP), understory vegetation diversity (Shannon index, Pielou index, species richness), soil microbial community diversity indices (Shannon index and Chao index), soil microbial community compositions and the relative abundance of the genes associated with different functional categories. Partial least squares discriminant analysis (PLS-DA), a supervised method, was performed on OTU data to discriminate the microbial community profiles of the four thinning treatments (Pérez-Enciso and Tenenhaus, 2003; Ma et al., 2017). Biomarker analysis was performed by linear discriminant analysis (LDA) for effect size (LEfSe) using the Kruskal–Wallis test and All-Against-All (more strict) to determine the significance of differences in soil microbial species among the four thinning treatments. LDA was performed to evaluate the difference in each microbial taxon with a threshold value of 3.5. Redundancy analysis (RDA) was used to test the relationships of microbial groups with understory vegetation and soil properties using Monte Carlo permutations (999 repetitions). Rarefaction curves, PLS-DA, correlation heatmap, and RDA were conducted with R 4.2.0 software.

## 3. Result

### 3.1. Understory vegetation and soil properties

The Shannon index, Pielou index and species richness of the shrub layer under the light-(LIT), moderate-(MIT) and high-(HIT) intensity thinning treatments were remarkably higher than those under the control (*p* < 0.05). While significant difference was absent in the herb layer among the four treatments (*p* ≥ 0.05) (Table 2).

**Table 2:** Understory vegetation and soil properties in *Cryptomeria japonica* var. *sinensis* plantations under four thinning treatments.

		Control	LIT	MIT	HIT
Herb	Shannon index	1.12 ± 0.20a	0.77 ± 0.75a	1.28 ± 0.13a	0.61 ± 0.61a
	Pielou index	0.87 ± 0.05a	0.45 ± 0.29a	0.74 ± 0.07a	0.45 ± 0.31a
	species richness	4 ± 0.58a	5 ± 3.00a	6 ± 0.58a	3 ± 1.53a
Shrub	Shannon index	0.21 ± 0.37b	1.21 ± 0.51a	1.51 ± 0.20a	1.35 ± 0.03a
	Pielou index	0.31 ± 0.53a	0.97 ± 0.05a	0.95 ± 0.03a	0.97 ± 0.02a
	species richness	1 ± 1.15b	4 ± 1.53a	5 ± 1.00a	4 ± 0.00a
SM (%)		0.12 ± 0.04b	0.27 ± 0.08a	0.15 ± 0.04b	0.25 ± 0.02a
SBD (g·cm^−3^)		1.32 ± 0.06a	1.03 ± 0.09c	1.18 ± 0.07b	1.03 ± 0.03c
pH value		4.26 ± 0.04a	4.03 ± 0.01c	4.14 ± 0.00b	4.01 ± 0.01c
SOM (g·kg^−1^)		32.98 ± 3.78a	34.48 ± 7.75a	41.16 ± 3.26a	40.04 ± 3.74a
TN (g·kg^−1^)		2.64 ± 0.00c	3.57 ± 0.01b	4.08 ± 0.24a	3.51 ± 0.00b
TK (g·kg^−1^)		10.81 ± 0.08b	12.14 ± 0.04a	11.92 ± 0.08a	8.21 ± 0.61c
TP (g·kg^−1^)		0.55 ± 0.01a	0.25 ± 0.00b	0.24 ± 0.00b	0.21 ± 0.02c
AN (mg·kg^−1^)		581 ± 49.00a	469.33 ± 147.00a	529.67 ± 91.34a	492.33 ± 42.19a
AK (mg·kg^−1^)		36.09 ± 1.52b	49.01 ± 2.29a	39.07 ± 7.05b	19.20 ± 3.19c
AP (mg·kg^−1^)		2.19 ± 0.09b	6.44 ± 0.64a	2.96 ± 0.18b	6.07 ± 0.49a

The values are the mean ± standard deviation (*n* = 3), followed by different letters showing significant differences in the four thinning treatments for each index at the 0.05 probability level. SM, soil moisture; SBD, soil bulk density; SOM, soil organic matter content; TN, TP, TK, AN, AK and AP, contents of total nitrogen, total phosphorus, total potassium, available nitrogen, available potassium, and available phosphorus, respectively.

Thinning significantly influenced the soil moisture (SM), soil bulk density (SBD), pH value, and contents of total nitrogen (TN), total potassium (TK), total phosphorus (TP), available potassium (AK) and available phosphorus (AP) (Table 2). The pH value, SBD and TP were significantly higher in the control than in the LIT, MIT and HIT treatments. The SM and TK in the LIT treatment were significantly higher than those in the control, MIT and HIT treatments. The TN was significantly higher in the MIT treatment than that in other treatments. The AP of LIT and HIT was higher than that of the control and MIT treatments. Significant differences were absent in the contents of soil organic matter (SOM) and available nitrogen (AN) among the four thinning treatments.

### 3.2. Soil microbial community diversity and composition

**Figure 1:**
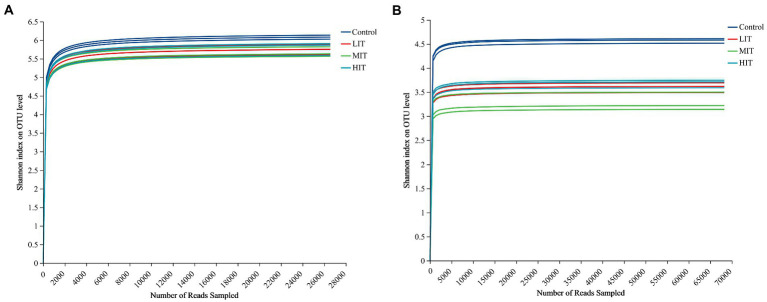
Rarefaction curve of Shannon index of bacteria **(A)** and fungus **(B)** communities at OTU level among the four thinning treatments.

A total of 318,732 bacterial and 819,288 fungal sequence reads were obtained from the complete dataset of 12 samples and were clustered into 3,994 and 2,807 OTUs, respectively. The rarefaction curve for thinning treatments of Shannon index on OUT level had been well captured at the amount of randomly drawn sequencing data (Figure 1). The Shannon and Chao indices of the bacterial community under the four thinning treatments ranged from 5.677 to 6.101 and from 2,485 to 3,110, respectively, and both indices of the fungal community ranged from 3.287 to 4.571 and from 1,028 to 1,402, respectively (Table 3). All of them were strongly influenced by thinning.

**Table 3:** Diversity indices of the bacterial and fungal communities among the four thinning treatments in *C. japonica* var. *sinensis* plantations.

		CK	LIT	MIT	HIT
Bacteria	Shannon	6.101 ± 0.058a	5.755 ± 0.13b	5.677 ± 0.134b	5.805 ± 0.175b
	Chao	3110.6 ± 163.91a	2523.5 ± 122.54b	2485.2 ± 238.61b	2837.6 ± 230.65ab
Fungi	Shannon	4.571 ± 0.05a	3.602 ± 0.103bc	3.287 ± 0.187c	3.684 ± 0.082b
	Chao	1402.2 ± 142.03a	1051.7 ± 50.09bc	1028.3 ± 63.51c	1135.5 ± 19.38b

The values are the mean ± standard deviation (*n* = 3), followed by different letters showing significant differences in the four thinning treatments for each index at the 0.05 probability level.

**Figure 2:**
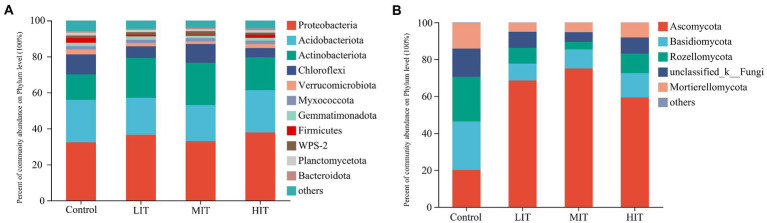
Relative abundance of the dominant groups of bacterial **(A)** and fungal **(B)** communities at the phylum level among the four thinning treatments.

The top three bacterial phyla in the bacterial community were *Proteobacteria* (relative abundance: 34.93%), *Acidobacteria* (21.82%), and *Actinobacteria* (19.57%) (Figure 2A). *Rhizobiales* (17.21%) and *Gammaproteobacteria_Incertae_Sedis* (2.1%) were the most abundant orders observed within the *Alphaproteobacteria* and *Gammaproteobacteria* classes, respectively (Supplementary Table S1). Significant differences were mostly absent in the relative abundances of *Proteobacteria*, *Acidobacteria* and *Verrucomicrobiota* among the four treatments, while the relative abundance of *Actinobacteriota* in the LIT, MIT and HIT treatments was significantly higher than that in the control (14.3 ± 3.25%) (Figure 2A).

*Ascomycota* (55.69%), *Basidiomycota* (14.72%), and *Rozellomycota* (11.85%) represented most of the observed fungal phyla (Figure 2B). The relative abundances of *Ascomycota* in the LIT (75.05 ± 3.89%) and MIT (86.46 ± 7.40%) treatments were significantly higher than those in the HIT (59.28 ± 2.75%) and control (20.06 ± 2.62%) treatments (Figure 2B). The relative abundances of *Basidiomycota*, *Rozellomycota* and *Mortierellomycota* in the control were 23.33 ± 2.05%, 24.10 ± 1.51% and 13.76 ± 2.89%, respectively, and were all significantly higher than those in the LIT, MIT and HIT treatments (Figure 2B).

**Figure 3:**
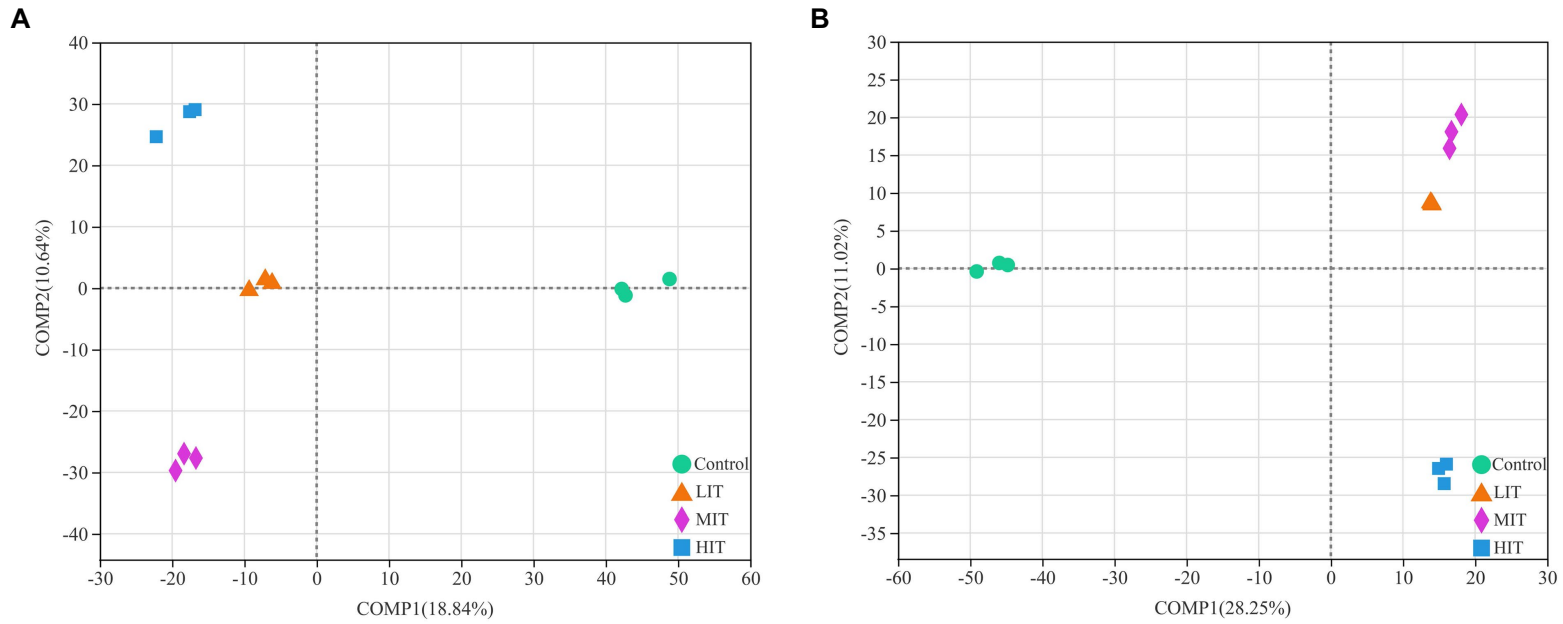
Partial least squares discriminant analysis (PLS-DA) of bacterial **(A)** and fungal **(B)** community composition among four thinning intensity treatment sites.

The PLS-DA analysis illustrated that the bacterial communities of the four thinning treatments were clearly separated from each other (Figure 3A), and the same were the fungal communities under the control and HIT treatments, while those under the LIT and MIT treatments were clustered together (Figure 3B).

The LEfSe algorithm was used to determine the taxa in the soil microbial communities (Supplementary Figure S1). In total, 22 bacterial clades presented statistically significant differences (LDA > 3.5, *p* < 0.05) in all soil samples. There were six differentially abundant taxa in the control (Supplementary Figure S1a), and the most important contribution to the control was the phylum *Firmicutes*, which accounted for 83%. The LIT treatment had the fewest biomarkers, with only two abundant bacterial clades. For the MIT treatment, the greatest contribution was made by four biomarkers, all of which came from the phylum *Firmicutes*, including the class *Actinobacteria,* with LDA scores of 4.50. The HIT treatment contained 17 biomarkers, and the categories with the highest contribution were mainly *Acidimicrobia*, *Planococcaceae*, *Chitinophagales*, and *Rhodospirillales*.

For fungi, 73 clades were determined by the LEfSe algorithm, which exhibited significant differences under four thinning treatments with an LDA threshold of 3.5 (Supplementary Figure S2). The control accounted for 72.6% of the fungal clades, while the LIT, MIT and HIT treatments had one, 15 and four clades, respectively. The proportion of the phylum *Rozellomycota* was highest in the control, followed by *Basidiomycota* (phylum) and *Tremellomycetes* (class). Specifically, the LIT treatment was only rich in *Meripilaceae* (family). The clades of the MIT and HIT treatments were mainly concentrated in *Ascomycota*.

Overall, the soil microorganisms composition was significantly different among the four thinning treatments, which to some extent reflects the impact of thinning on the soil microbial community.

### 3.3. Relationships of microbial communities with understory vegetation and soil properties

**Figure 4:**
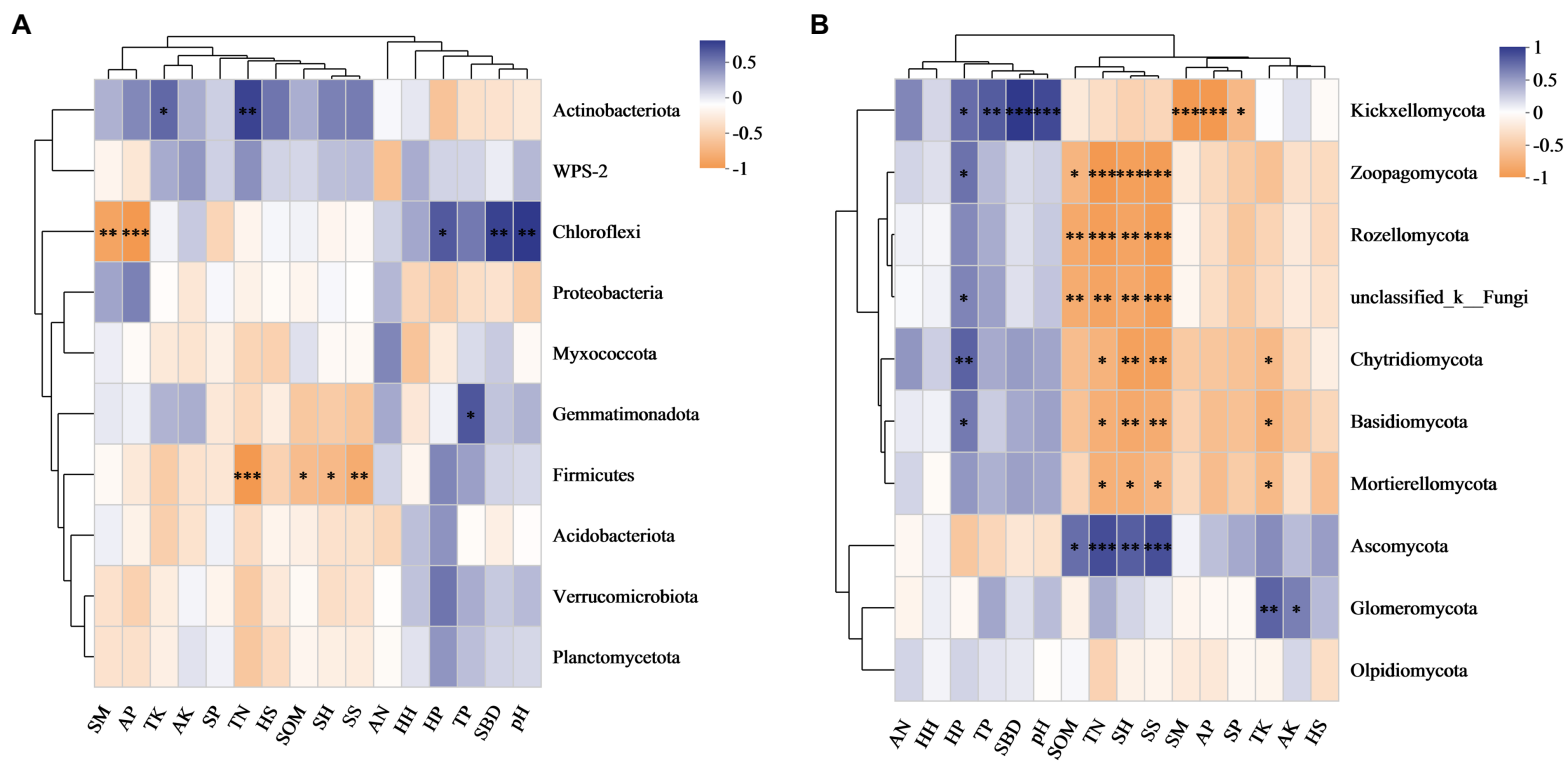
Correlation heatmaps of the abundance of bacterial **(A)** and fungal **(B)** communities at the phylum level with understory vegetation characteristics and soil properties. HH, HP, HS, SH, SP and SS denote the Shannon index, Pielou index and species richness in the herb and shrub layers, respectively. SM, soil moisture; SBD, soil bulk density; SOM, soil organic matter content; TN, TK, TP, AN, AK and AP, contents of total nitrogen, potassium, phosphorus, available nitrogen, potassium and phosphorus, respectively.

**Figure 5:**
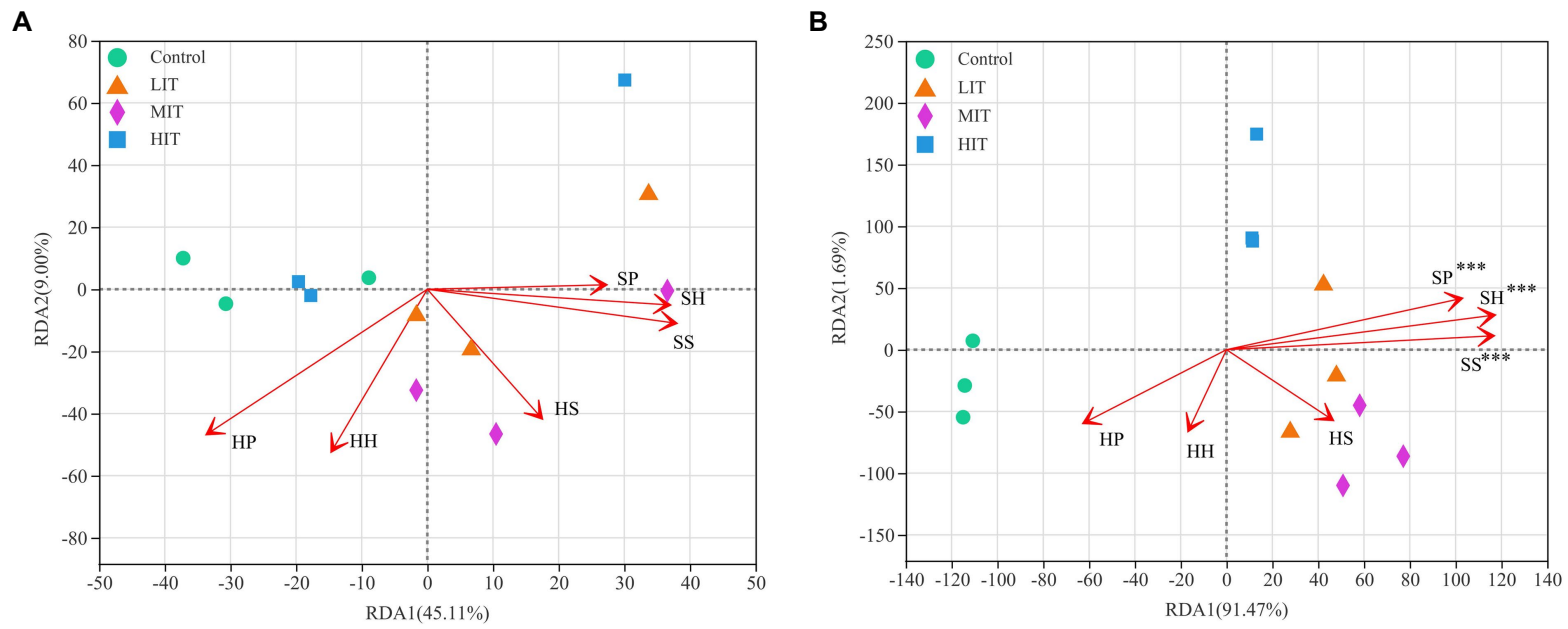
RDA of abundant bacterial **(A)** and fungal **(B)** communities at the genus level and understory vegetation characteristics for the soil samples from four thinning treatments in *Cryptomeria japonica* var. *sinensis* plantations. HH, HP, HS, SH, SP and SS denote the Shannon index, Pielou index and species richness in the herb and shrub layers, respectively. *p < 0.05, **p < 0.01, ***p < 0.001.

The Pielou index in the herb layer was positively correlated with microbes, especially fungi (Figure 4). The Shannon index and species richness in the shrub layer were significantly negatively correlated with the relative abundances of *Zoopagomycota*, *Rozellomycota*, *Chytridiomycota*, *Basidiomycota* and *Mortierellomycota* (Figure 4B). According to the redundancy analysis (RDA), the total interpretation degree of the understory vegetation index to the bacterial genus level was 54.11% (Figure 5A), and three indicators in the shrub layer showed a significant correlation with fungal community composition (Figure 5B).

SBD and pH value were significantly and positively correlated with the relative abundance of *Chloroflexi*, while SM and AP were significantly and negatively correlated with it. The relative abundance of *Firmicutes* was negatively correlated with TN and SOM (Figure 4A). SOM and TN were significantly and positively correlated with the relative abundance of *Ascomycota*. They were significantly and negatively correlated with the relative abundances of *Zoopagomycota*, *Rozellomycota*, *Chytridiomycota*, *Basidiomycota* and *Mortierellomycota*, respectively (Figure 4B).

**Figure 6:**
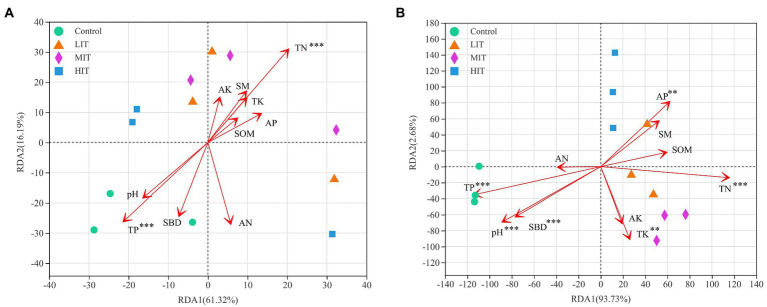
RDA of abundant bacterial **(A)** and fungal **(B)** communities at the genus level and soil properties for the soil samples from four different thinning treatments in *C. japonica* var. *sinensis* plantations. SM, soil moisture; SBD, soil bulk density; SOM, soil organic matter; TN, TK, TP, AN, AK, and AP, contents of total nitrogen, total potassium, total phosphorus available nitrogen, available potassium and available phosphorus, respectively. *p < 0.05, **p < 0.01, ***p < 0.001.

Figure 6A shows that the first and second ordination axes of RDA explained 61.32 and 16.19% of the variance in the soil bacterial community, respectively, and TP and TN explained the largest proportion of the variance. The pH value, SBD, TN, TK, TP and AP appeared to be the most important environmental parameters for the fungal community, and the variance explained by the first and second ordination axes was 93.73 and 2.68%, respectively (Figure 6B).

### 3.4. Microbial function

A total of 40 groups at level 2 of KEGG orthologs (KO) were detected (Supplementary Table S2). There were 6,053 KOs across all samples, and the most abundant functional pathways were presented in Supplementary Figure S3. The abundances of metabolism, environmental information processing and genetic information processing accounted for a relatively high proportion of the four thinning treatments, with mean values of 60.07%, 22.04%, and 10.03%, respectively. Significant differences were found in bacterial functional pathways among the thinning treatments. Analysis of level 2 metabolic pathways showed that the abundances of bacterial functions such as membrane transport, signal transduction, energy metabolism, and cell motility increased significantly with increasing thinning intensity.

**Figure 7:**
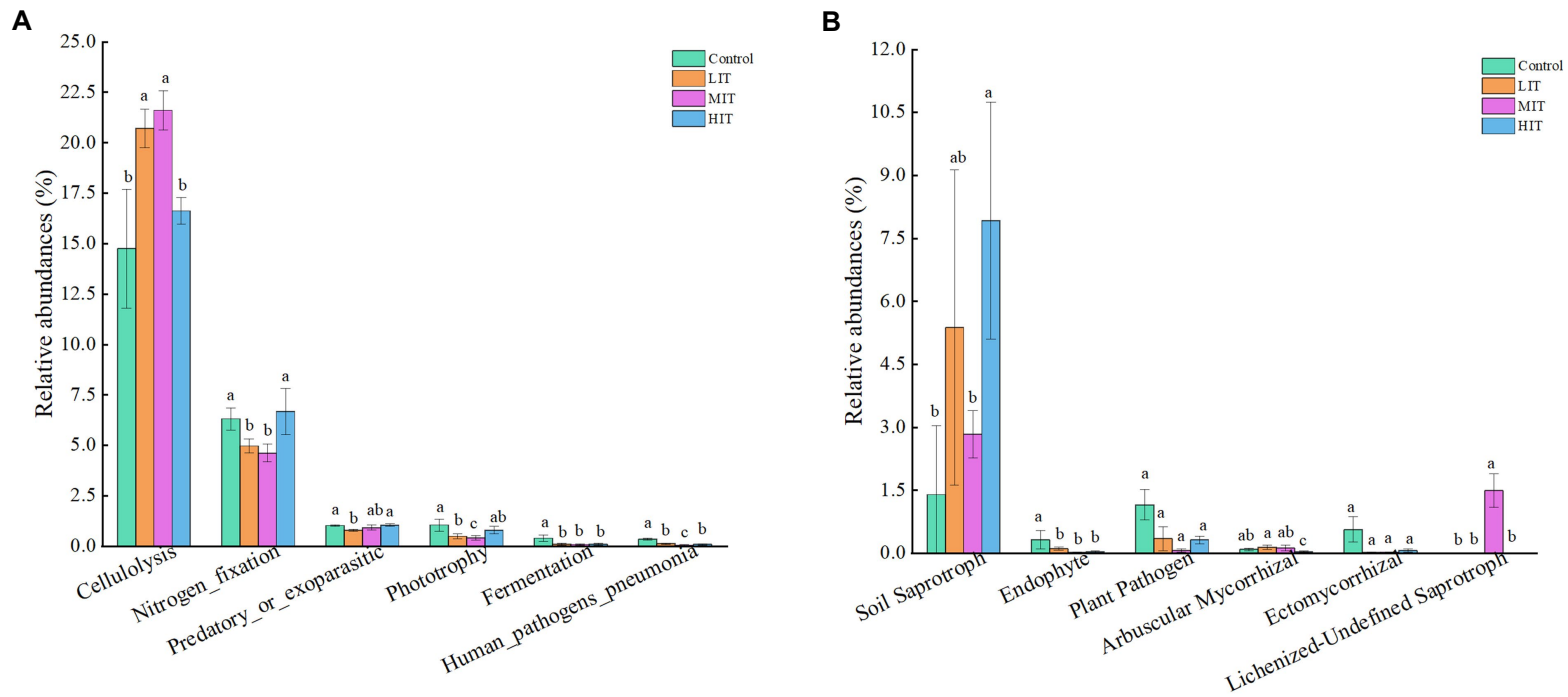
Variations in the composition of bacterial **(A)** and fungal **(B)** functional groups under different thinning treatments. The bars represent standard deviation of the means (*n* = 3); different lowercase letters indicate significant differences between different thinning intensities in the same index.

The abundances of nucleotide metabolism, translation, replication, and repair were opposite to the abovementioned changes (Supplementary Table S2). The proportion of cellulolysis was significantly increased in the LIT, MIT and HIT treatments. The relative abundances of nitrogen fixation and phototrophy in the LIT and MIT treatments were lower than those in the control (Figure 7A).

Most fungi could not be classified (classification = ‘Unknown’), and 56.27%, 81.46%, 82.37%, and 70.91% of the relative abundance of fungal functional groups were unclassified in the control, LIT, MIT and HIT treatments, respectively (Supplementary Figure S4). The most abundant fungal parasite, undefined saprotrophs, had a mean abundance of 14.26% in the control, almost three times as much as it did in the LIT or MIT treatment (Supplementary Figure S4). Thinning significantly enhanced the abundance of soil saprotrophs and lichenized-undefined saprotrophs, and the abundance of soil saprotrophs in HIT was 5 times higher than that in the control (Figure 7B). The abundance of endophytes, plant pathogens and ectomycorrhizal fungi decreased with increasing thinning intensity.

## 4. Discussion

### 4.1. Response of understory vegetation diversity and soil properties to thinning intensities

In the present study, the Shannon index, the Pielou index and the species richness of the shrub layer in the HIT, MIT and LIT treatments were notably higher than those in the control. The main reason is that thinning results in more or larger canopy gaps and improves understory light conditions, which increases plant diversity (Tamura and Yamane, 2017). However, the effect of thinning on the richness of the herb layer was not as obvious as those in previous reports (Son et al., 2004; Muscolo et al., 2021), and the species richness was greater in the shrub layer than in the herb layer. These results were presumably related to the fact that shrubs are taller than herbaceous plants and can embrace light first, as the amount of light reaching the understory is one of the most important limiting factors affecting the species of understory plants (Yu et al., 2022). Additionally, the lack of seed resources of understory vegetation species in and around the plot due to the relatively high tree density before thinning (Ali et al., 2019b) and the altitude of the site might be another reason for the lower number of vegetation species.

Understory vegetation cover and richness increased with increasing thinning intensities in the present, which led to an increase in the amount of litter (He and Barclay, 2000; Teste et al., 2012), followed by changes in soil physical attributes and nutrient cycling (Elliott et al., 2015; Xiao et al., 2018; Yang et al., 2019). The roots and dead parts of the newly occurred herbs and shrubs are rich in lignin and other substances that enhance the looseness of the topsoil layer (Osman, 2013), which was the main reason why the soil bulk density decreased as the thinning intensity increased in the present study. Then, the humification of litter increased the contents of soil organic acids and organic matter (Sayer, 2006; Zou et al., 2017). The soil pH value difference in the present study was related to the changes in soil organic acid generation activity after thinning, and soil nutrients, especially the contents of soil organic matter (SOM) and total nitrogen (TN) were increased after thinning. Compared with the control, the SOM and TN under the high-intensity thinning treatment (HIT) increased by 19.87% and 35.29%, respectively (Table 2). Thus, it can be seen that LIT and MIT treatments were conducive to improving the understory vegetation diversity and soil physicochemical properties of young *C. japonica* var. *sinensis* plantation.

### 4.2. Responses of soil microbial community diversity and composition to thinning intensities

With increasing thinning intensities, the Shannon and Chao indices of soil bacteria and fungi first decreased and then increased in the *C. japonica* var. *sinensis* forest (Table 3). This was different from the study of Dang et al. (2018), in which the Shannon and Chao indices in soil bacteria and fungi did not vary significantly with thinning intensities. Cai et al.’s (2020) study on the thinning of *Larix gmelinii* var. *principis-rupprechtii* plantation showed that the Shannon index of bacterial communities were higher in the medium-intensity than in other treatment. Lin et al. (2016) demonstrated that soil fungal communities did not change significantly after 21 months of thinning in a Japanese cedar (*Cryptomeria japonica*) plantation in Taiwan. The reason for inconsistent results among these studies may be related to the difference in species composition and distribution pattern of the understory vegetation since microbial community composition varies with vegetation species (Overby et al., 2015). Thinning promotes the growth of planted trees and understory vegetation, which is the main reason for changing the composition and decomposition rate of litter and therefore affects the activity of soil microorganisms. This was also demonstrated in a study of mature *Pinus contorta* forests, which found that the heterogeneity of understory plant species and their rhizosphere resources (e.g., root exudates, nutrients) influenced the patterns of variation in belowground microbial communities (McIntosh et al., 2013). In addition, the changes in the soil bacterial and fungal communities may be related to their adaptability to the changed microenvironment in the stands after thinning.

Thinning significantly changed the soil bacterial community structure in the *C. japonica* var. *sinensis* plantation at the phylum level, and the relative abundances of *Proteobacteria* and *Actinobacteriota* increased with increasing thinning intensity (Figure 2A). This could be explained by the enhanced soil nitrogen content under thinning treatments (Table 2), as in Wang et al.’s (2018) study on Chinese fir (*Cunninghamia lanceolata*) plantations, in which nitrogen addition could increase the relative abundance of *Proteobacteria* and *Actinobacteria*. The study of Cai et al. (2020) showed that these two phyla had higher abundance in the no thinning treatment of *Larix* plantations. The most reasonable explanations are that the bacterial composition is closely related to the soil conditions, climate and sampling time (Green et al., 2008), and the adaptability of soil microorganisms in the community to soil environmental changes is different (Navarrete et al., 2015; Ren et al., 2016). Accordingly, the phylum level of bacteria may respond differently to changes in forest density. This study found that soil bacterial communities at the phylum level were mainly composed of *Proteobacteria* (relative abundance 34.93%) in four treatments (Figure 2A), and the second dominant phylum was *Acidobacteriota*, followed by *Actinobacteriota* and *Chloroflexi*. Coincidentally, the LEfSe algorithm showed that *Bacilli* (*Firmicutes*) may be a potential biomarker of bacteria in the control (Supplementary Figure S1). With increasing thinning intensities, *Gammaproteobacteria* (*Proteobacteria*) and *Acidimicrobia* (*Actinobacteriota*) may be potential biomarkers for light-intensity (LIT) and high-intensity (HIT) thinning treatments, respectively. For moderate-(MIT) intensity thinning treatments, *Actinobacteriota* and *Chloroflexi* may be potential biomarkers. *Proteobacteria* show a significant positive correlation with the soil organic carbon concentration, indicating that increases in root secretions or plant litter decomposition products stimulate their growth (Huhe et al., 2017). *Actinobacteria* species are recognized as degraders of cellulose, chitin and other complex carbon compounds, which gives them a central role in the carbon cycle and in the turnover of organic matter (Yergeau et al., 2010; Zhou et al., 2017). *Acidobacteria* and *Chloroflexi* also play an important role in the decomposition of organic matter and nutrient cycling (Bryant and Frigaard, 2006; Eichorst et al., 2018). *Firmicutes* can reduce the abundance of plant pathogens (Ali et al., 2019a), and they also embrace sulfate- and iron- reduction abilities (Gupta et al., 2018). These results demonstrate that changes in soil bacterial community composition are directly related to soil characteristics, especially changes in carbon and nitrogen.

The abundance of *Ascomycota* increased significantly, while *Basidiomycota*, *Rozellomycota*, and *Mortierellomycota* decreased at the soil fungal phylum level with increasing thinning intensities (Figure 2B). *Ascomycota* is a potential biomarker of the LIT, MIT and HIT treatments, and as the most abundant fungus, *Ascomycota* accounted for up to 75% (MIT treatment) of fungi in the *C. japonica* var. *sinensis* plantation, in which *Archaeorhizomycetes* had the highest abundance (Supplementary Table S1), and increased with increasing thinning intensities*. Archaeorhizomycetes* are a type of saprophytic fungi (Rosling et al., 2011) that play an important role in the cycling of carbon derived from living or dead roots in soil (Rosling et al., 2013). The LEfSe algorithm showed that fungi had the most taxonomic groups in the control, which were 53, 3.53 and 13.25 times that of the LIT, MIT and HIT treatments, respectively. *Rozellomycota*, *Basidiomycota*, and *Tremellomycetes* (*Basidiomycota*) may be potential biomarkers for the control (Supplementary Figure S2). *Basidiomycota* are found in almost all terrestrial environments, and many of them obtain nutrients by decaying wood, leaf litter and other dead organic matter. Therefore, they play an indispensable role in the carbon cycle. *C. japonica* var. *sinensis* had dense branches with a dark and moist environment in the control plots, and it was easy to cause the bottom branches to wither away and fall down. Branches of conifer species are more difficult and need a longer time to be decomposed than shrubs and herbs, and the present study was conducted 5 years after thinning, which may be the reason for the higher abundance of *Basidiomycota* in the control.

### 4.3. Relationships of microbial communities with understory vegetation and soil properties

The relationship of microbial community structure with understory vegetation and soil is one of the vital goals of microbial ecology (van der Heijden et al., 2008; Fu et al., 2018). In this study, the Pielou index of the herb layer was positively correlated with the abundance of *Chloroflexi*, *Planctomycetota* and *Verrucomicrobiota*, and was negatively correlated with the abundance of *Actinobacteria* (Figure 4A). The Shannon index and species richness in the herb layer and the Shannon index, Pielou index and species richness in the shrub layers were almost negatively correlated with the abundance of the top 10 bacterial phyla, and the bacterial community was more likely to be significantly correlated with the soil properties than vegetation. This demonstrated that soil physicochemical properties had more important effects on bacterial community structure than did understory vegetation diversity. We also found that the pH value and soil bulk density (SBD) were significantly positively correlated with the relative abundance of *Chloroflexi*. One study on the depth profiles of microbial communities in high-elevation soils demonstrated that soil pH was an important driver of forming soil bacterial communities in a given region (Chu et al., 2016). However, Dang et al. (2018) revealed that pH was not correlated with dominant members of bacterial communities in *Pinus tabuliformis* Carriere plantations. The SM and available phosphorus content (AP) were the main driving factors of the soil bacterial community structure in the *C. japonica* var. *sinensis* plantation; they were positively correlated with the abundance of *Proteobacteria* and negatively correlated with the abundance of *Chloroflexi*. *Proteobacteria* can improve soil fertility and sustainability (Rousk et al., 2009; Niu et al., 2020). *Chloroflexi* is slow-growing and was once classified as an oligotrophic group, and its growth and development may be limited by soil nutrient accumulation (Xu et al., 2021; Zhang et al., 2022), which is why TN, AP and the content of total potassium (TK) were negatively correlated with the abundance of *Chloroflexi*.

The correlation heatmap of understory vegetation diversity and soil properties with the main phylum of fungi showed that the relative abundances of the main phylum fungi, except *Ascomycota* and *Glomeromycota*, were negatively correlated with the indices of the shrub layer and positively correlated with SBD, pH value and the content of total phosphorus (TP). RDA further confirmed that SBD, pH value, TK, TP, TN, AP and shrub diversity affected the abundance of fungal communities. Overall, soil parameters were the most important factors affecting the soil fungal community structure. This was in accordance with Adamo et al.’s (2021) study in 42 pure and mixed pine forests, which showed that soil chemistry significantly affected the variability of soil fungal communities.

Thinning had significant effects on the relative abundances of the dominant fungal communities rather than the dominant bacterial communities, which might indicate that the bacterial community has stronger resistance to changes in stand density than fungal communities (Wang C. Q. et al., 2021). Some studies have also reported that fungal communities have a more obvious response to plant–soil feedback than bacterial communities (Dang et al., 2018; Hou et al., 2021). This is mainly because the bacterial community has a smaller ecological niche in soil and a weaker symbiotic relationship with plants than the fungal community (Shan et al., 2017). Therefore, changes in soil properties, directly or indirectly caused by the intensity of thinning, were less responsive to bacterial than to fungal community diversity.

### 4.4. Potential metabolic pathways in soils

Understory vegetation diversity and contents of soil nutrients in the *C. japonica* var. *sinensis* plantation increased with increasing thinning intensities. Different bacterial and fungal species had different strategies for adaptation to microenvironmental changes in the thinned plantations. Analysis of microbial function prediction showed that 60% of bacteria were involved in metabolic pathways, some of which are known to cause human disease, and the remaining bacteria were involved in genetic information processing, environmental information processing, organismal systems and cellular processes. The abundances of membrane transport, nucleotide metabolism and signal transduction in the LIT, MIT and HIT plots were higher than those in the control. Bacteria often have a strong relationship with human diseases (Geng et al., 2020; Wang et al., 2020). In this study, bacterial infectious diseases accounted for the highest proportion of human diseases, up to 10%. Fortunately, the proportion of human diseases decreased with increasing thinning intensities. This inferred that the change in the bacterial community caused by thinning in the *C. japonica* var. *sinensis* plantations might reduce the possibility of harm to humans.

Thinning significantly increased the functional groups ‘soil saprotrophs’ while decreasing the functional groups ‘endophytes’ and ‘plant saprotrophs’ in fungi. This proved that soil nutrients under the *C. japonica* var. *sinensis* plantation could be improved by thinning since the abundance of soil saprotrophs was positively correlated with soil fertility (Kyaschenko et al., 2017). This was further confirmed by RDA, from which a significant positive correlation was found between soil properties and the fungal community. Based on the composition and community function of soil bacteria and fungi, it has been predicted that higher thinning intensities are beneficial for soil properties and soil microbial relationships in *Cryptomeria japonica* var. *sinensis* plantations.

## 5. Conclusion

This study showed that thinning enhanced the understory vegetation diversity and improved the soil physicochemical properties. In particular, thinning had a greater effect on the diversity of the shrub layer than the herb layer, and the soil bulk density (SBD) and the contents of soil organic matter (SOM) and total nitrogen (TN) increased with increasing thinning intensities of *Cryptomeria japonica* var. *sinensis* plantations. Although the Shannon and Chao indices of soil bacteria and fungi were significantly lower in the HIT, MIT and LIT treatments, the abundance of soil bacterial and fungal species varied significantly with different thinning intensities, the abundance of *Actinobacteriota* and *Ascomycota* significantly increased, and the abundance of *Basidiomycota*, *Rozellomycota* and *Mortierellomycota* decreased with increasing thinning intensities. The effects of thinning on microorganisms were mainly driven by soil properties such as pH value and the contents of total nitrogen and total phosphorus, especially for fungi. Fungi are more sensitive to understory vegetation than bacteria. Changes in the distribution of microbial function are a response to changes in the microbial community composition. Thinning improves membrane transport, signal transduction, and cellulolysis in bacteria and soil saprotrophs in fungi. According to the changes of understory vegetation diversity, soil physicochemical properties and microbial composition and function after thinning, LIT and MIT treatments should be adopted in young *C. japonica* var. *sinensis* plantations. This study illustrated the relationship of soil microorganisms with the understory vegetation and soil properties in plantations with different thinning densities; however, the effect of thinning on plant–soil-microorganism interactions in plantations needs to be further studied to explore the interaction mechanisms among them.

## Data availability statement

The datasets presented in this study can be found in online repositories. The names of the repository/repositories and accession number(s) can be found below: NCBI—PRJNA928327.

## Author contributions

K-LL: investigation, data curation, software, formal analysis, writing—original draft, and writing—review and editing. B-YC: investigation, data curation, and visualization. BZ: conceptualization, methodology, and investigation. R-HW: conceptualization, methodology, supervision, funding acquisition. C-SW: methodology, software, formal analysis, writing—review and editing, and supervision. All authors contributed to the article and approved the submitted version.

## Conflict of interest

The authors declare that the research was conducted in the absence of any commercial or financial relationships that could be construed as a potential conflict of interest.

## Publisher’s note

All claims expressed in this article are solely those of the authors and do not necessarily represent those of their affiliated organizations, or those of the publisher, the editors and the reviewers. Any product that may be evaluated in this article, or claim that may be made by its manufacturer, is not guaranteed or endorsed by the publisher.

## Footnotes

^1^ https://github.com/OpenGene/fastp

^2^ https://ccb.jhu.edu/software/FLASH/index.shtml

^3^ http://www.drive5.com/uparse/

^4^ http://www.arb-silva.de

^5^ http://unite.ut.ee/index.php

^6^ http://sourceforge.net/projects/rdp-classifier/

^7^ https://github.com/UMNFuN/FUNGuild
