# Supplementary material for: Understory vegetation diversity, soil properties and microbial community response to different thinning intensities in Cryptomeria japonica var. sinensis plantations
Source: Front Microbiol. 2023 Feb 28;14:1117384. doi: 10.3389/fmicb.2023.1117384 (PMC10011715; doi:10.3389/fmicb.2023.1117384)
Supplement: Supplementary file 1 [file Data_Sheet_1.PDF]

Soil properties rather than understory vegetation diversity driving microbial community changes after thinning in *Cryptomeria japonica* var. *sinensis* plantations

Supplementary data

Table S1

Relative abundances (average values >= 1% and standard deviations) of dominant bacterial and fungal order groups in the soils of the four thinning treatments of *Cryptomeria japonica* var. *sinensis* plantations. The effect of thinning treatments on relative abundance was evaluated by One-way analysis of variance (ANOVA). * *P value* < 0.05, ** *P value* < 0.01, *** *P value* < 0.001. The values are the mean ± standard deviation (n=3), followed by different letters showing significant differences in the four thinning treatments for each index at the 0.05 probability level.

	CK	LIT	MIT	HIT	*F*	*P-value*
Bacterial						
o__Rhizobiales	15.66±0.62a	18.35±3.94a	15.76±2.44a	19.07±4.4a	0.898	0.483
o__Acidobacteriales	8.58±2.12a	5.74±2.49a	6.62±2.41a	6.95±3.76a	0.55	0.662
o__Subgroup_2	8.95±1.63a	8.41±3.46a	7.79±2.86a	9.23±4.74a	0.107	0.953
o__Ktedonobacterales	5.8±0.59a	3.16±1.38bc	5.07±1.59ab	2.97±1.7bc	3.076	0.091
o__Elsterales	6.4±2.21a	7.73±0.89a	7.42±1.29a	6.33±1.04a	0.718	0.569
o__Frankiales	6.26±1.74b	11.79±1.43a	12.5±1.58a	8.69±1.59b	9.921	0.005
o__Solibacterales	2.34±0.23a	2.37±0.46a	2.54±0.65a	2.72±0.89a	0.244	0.863
o__norank_c__AD3	2.63±0.49ab	1.48±0.36cb	3.48±1.22a	0.92±0.17c	8.34	0.008
o__Bryobacterales	1.97±0.1a	2.43±0.46a	2.21±0.52a	3.14±1.43a	1.194	0.372
o__Bacillales	1.74±0.3a	0.44±0.06c	0.28±0.08c	1.03±0.44b	17.933	0.001
o__Gammaproteobacteria_Incertae_Sedis	1.77±0.31b	2.45±0.06a	2.06±0.15ab	2.11±0.17ab	5.996	0.019
o__Gemmatimonadales	1.85±0.2a	1.89±0.36a	1.43±0.13b	1.47±0.23ab	3.022	0.094
o__Chlamydiales	1.22±0.43a	0.61±0.35a	0.62±0.29a	0.72±0.39a	1.805	0.224
o__IMCC26256	1.68±0.09a	2.24±0.61ab	2.39±0.23b	1.66±0.24a	3.581	0.066
o__Burkholderiales	1.44±0.1a	1.34±0.23a	1.11±0.32a	1.43±0.4a	0.877	0.492
o__norank_c__norank_p__WPS-2	1.38±0.31a	1.33±0.5a	1.91±0.73a	1.13±0.54a	1.143	0.389
o__Solirubrobacterales	2.46±1.17a	2.28±0.31a	2.66±0.85a	2.28±0.26a	0.17	0.914
o__Chthoniobacterales	1.02±0.24a	0.66±0.54a	0.36±0.22a	0.71±0.46a	1.428	0.305
o__Gemmatales	0.92±0.22a	0.59±0.41a	0.48±0.19a	0.71±0.49a	0.87	0.496
o__Vicinamibacterales	0.81±0.32a	0.84±0.34a	0.45±0.2a	0.69±0.36a	0.972	0.452
o__norank_c__Acidimicrobiia	1.27±0.27b	2.49±0.25a	2.91±0.28a	2.98±0.64a	12.108	0.002
o__Acetobacterales	0.94±0.15b	1±0.11ab	1.3±0.2ab	1.46±0.44a	2.816	0.107
o__Micropepsales	0.71±0.15a	0.87±0.14a	0.88±0.05a	0.98±0.29a	1.151	0.386
o__Polyangiales	0.91±0.12a	1.01±0.1a	1.07±0.15a	1.15±0.21a	1.336	0.329
o__norank_c__Alphaproteobacteria	0.91±0.18a	0.67±0.19ab	0.44±0.09b	0.6±0.12b	5.108	0.029

o__norank_c__TK10	0.72±0.1a	0.42±0.04b	0.44±0.09b	0.36±0.07b	12.714	0.002
o__Corynebacteriales	0.77±0.1ab	0.71±0.05b	0.99±0.26a	0.7±0.06b	2.475	0.136
o__norank_c__norank_p__RCP2-54	0.9±0.41a	0.86±0.12a	0.73±0.37a	0.62±0.16a	0.576	0.647
o__Gaiellales	0.85±0.13b	1.34±0.39a	0.93±0.11ab	0.86±0.1b	3.328	0.077
Fungi						
o__unclassified_c__Archaeorhizomycetes	0.91±0.28b	44.88±1.15a	50.79±4.41a	37.31±0.89a	278.137	0.000
o__unclassified_p__Ascomycota	2.21±0.4c	14.2±4.14a	18.38±1.06a	8.71±1.62b	28.035	0.000
o__Mortierellales	13.52±2.76a	4.84±0.87b	5.13±2.75b	8.02±3.65b	6.648	0.015
o__Tremellales	14.3±1.88a	4.6±1.03b	5.36±0.09ab	8.06±2.76ab	19.022	0.001
o__Filobasidiales	9.75±0.39a	3.89±1.04b	2.85±1.3b	3.63±1.31b	26.123	0.000
o__unclassified_k__Fungi	15.36±1.49a	8.77±3.3b	5.28±1.67b	8.79±1.68b	11.366	0.003
o__Archaeorhizomycetales	1.15±1.74b	5.36±3.75ab	2.83±0.56ab	7.89±2.83a	4.099	0.049
o__unclassified_p__Rozellomycota	17.17±0.91a	5.82±1.73b	3.26±1.01c	4.96±1.06bc	80.750	0.000
o__Agaricales	0.52±0.09b	0.07±0.04c	1.7±0.37a	0.2±0.09bc	43.350	0.000
o__Hypocreales	5.45±0.82a	1.5±0.47b	1.03±0.18b	1.58±0.11b	53.928	0.000
o__GS11	6.85±1.1a	2.66±0.89c	0.68±0.11d	5.43±0.45b	41.221	0.000

Table S2

Abundance of predicted KEGG categories under four thinning treatments.

		CK	LIT	MIT	HIT
Environmental Information Processing	Membrane transport	4.6571	4.7277	4.7389	4.7191
Metabolism	Nucleotide metabolism	2.4959	2.4607	2.4505	2.4660
Environmental Information Processing	Signal transduction	0.9795	0.9928	0.9931	0.9878
Metabolism	Energy metabolism	0.9430	0.9588	0.9631	0.9553
Metabolism	Amino acid metabolism	0.9075	0.9078	0.9075	0.9081
Metabolism	Carbohydrate metabolism	0.8381	0.8330	0.8349	0.8360
Genetic Information Processing	Translation	0.8323	0.8122	0.8086	0.8151
Cellular Processes	Cell motility	0.8295	0.8392	0.8420	0.8381
Metabolism	Metabolism of cofactors and vitamins	0.5672	0.5669	0.5676	0.5673
Genetic Information Processing	Replication and repair	0.5420	0.5284	0.5256	0.5304
Cellular Processes	Cell growth and death	0.5183	0.5265	0.5257	0.5228
Genetic Information Processing	Folding, sorting and degradation	0.4157	0.4076	0.4068	0.4092
Cellular Processes	Cellular community - prokaryotes	0.3800	0.3910	0.3874	0.3859
Metabolism	Metabolism of other amino acids	0.2872	0.2862	0.2851	0.2859
Metabolism	Xenobiotics biodegradation and metabolism	0.2311	0.2354	0.2357	0.2342
Metabolism	Lipid metabolism	0.2195	0.2180	0.2179	0.2184
Organismal Systems	Environmental adaptation	0.2054	0.2027	0.2005	0.2024
Metabolism	Glycan biosynthesis and metabolism	0.2005	0.1960	0.1958	0.1969
Metabolism	Metabolism of terpenoids and polyketides	0.1656	0.1637	0.1637	0.1644
Human Diseases	Infectious disease: bacterial	0.1098	0.1010	0.0980	0.1023
Cellular Processes	Transport and catabolism	0.0942	0.0948	0.0946	0.0944
Genetic Information Processing	Transcription	0.0575	0.0555	0.0553	0.0559
Metabolism	Biosynthesis of other secondary metabolites	0.0538	0.0536	0.0536	0.0537
Human Diseases	Cancer: overview	0.0472	0.0482	0.0480	0.0477

Organismal Systems	Endocrine system	0.0446	0.0449	0.0455	0.0450
Human Diseases	Neurodegenerative disease	0.0347	0.0340	0.0342	0.0342
Human Diseases	Endocrine and metabolic disease	0.0335	0.0339	0.0341	0.0339
Human Diseases	Infectious disease: parasitic	0.0257	0.0258	0.0256	0.0256
Organismal Systems	Nervous system	0.0170	0.0171	0.0170	0.0170
Organismal Systems	Circulatory system	0.0148	0.0152	0.0154	0.0151
Human Diseases	Substance dependence	0.0112	0.0117	0.0118	0.0116
Organismal Systems	Digestive system	0.0097	0.0090	0.0089	0.0091
Human Diseases	Cancer: specific types	0.0088	0.0088	0.0088	0.0088
Human Diseases	Immune disease	0.0082	0.0068	0.0064	0.0070
Organismal Systems	Immune system	0.0068	0.0061	0.0060	0.0063
Organismal Systems	Excretory system	0.0039	0.0039	0.0039	0.0039
Human Diseases	Infectious disease: viral	0.0023	0.0024	0.0024	0.0023
Human Diseases	Cardiovascular disease	0.0016	0.0016	0.0016	0.0016
Cellular Processes	Cellular community - eukaryotes	0.0004	0.0004	0.0003	0.0003
Environmental Information Processing	Signaling molecules and interaction	0.0002	0.0002	0.0002	0.0002

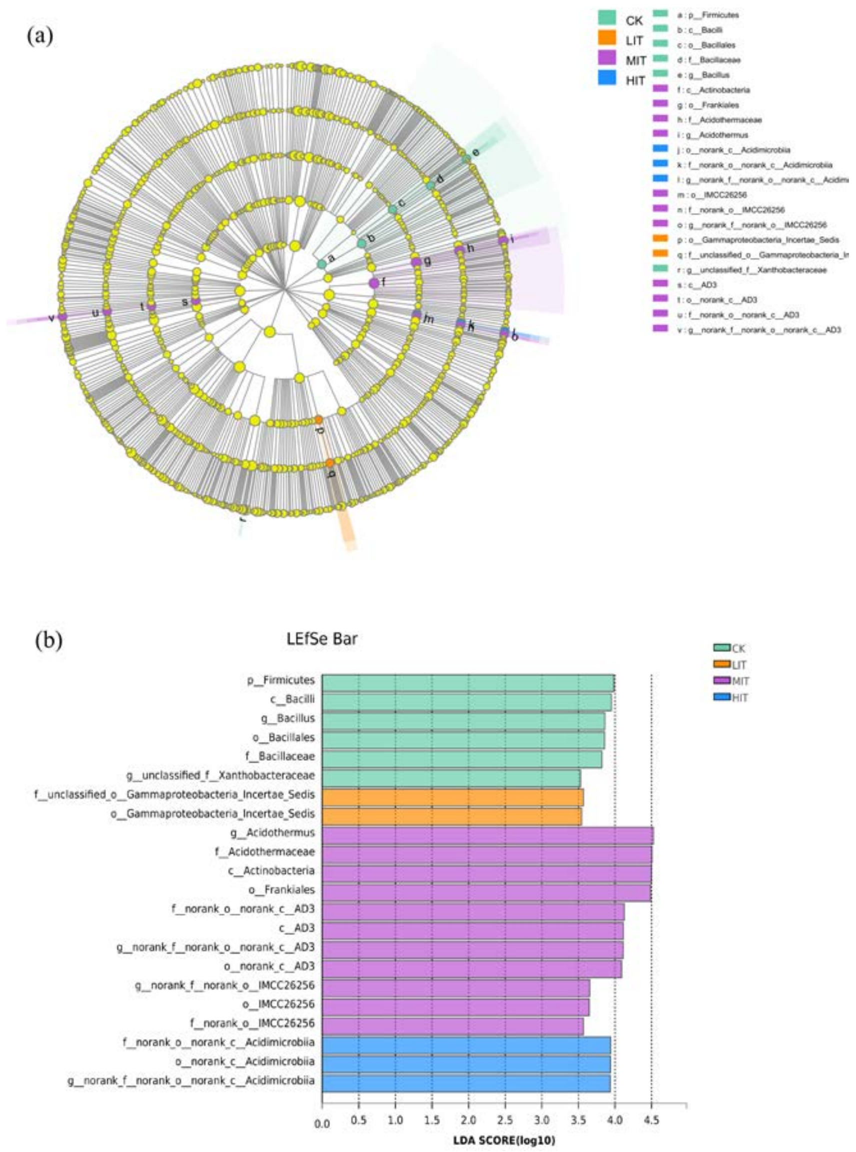

Fig. S1 LEfSe analysis of soil bacteria abundance in four thinning treatments: LEfSe analysis results of soil bacteria (a), and Histogram of LDA scores calculated for the differentially abundant bacteria with a threshold value of 3.5 (b).

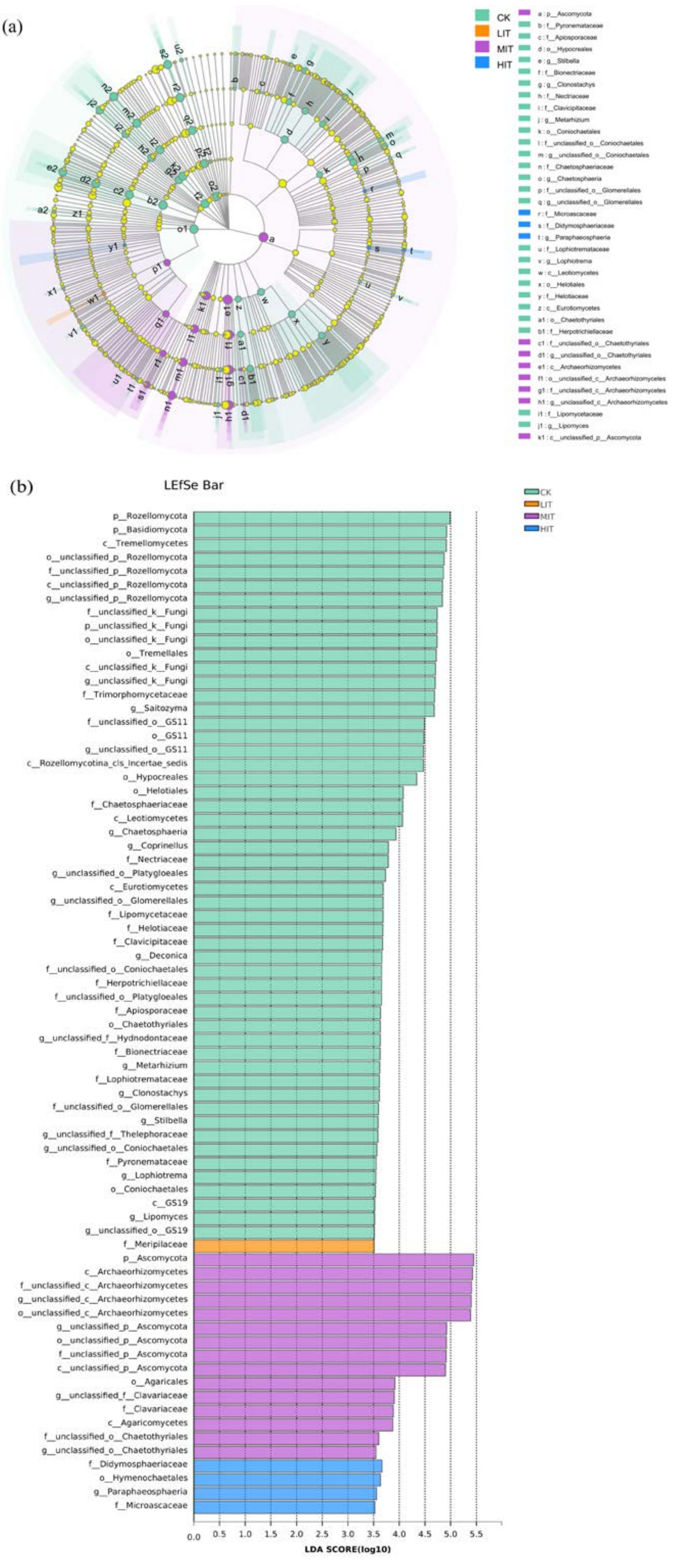
(a)
CK
LIT
MIT
HIT
a : p__Ascomycota
b : f__Pyronemataceae
c : f__Apiosporaceae
d : o__Hypocreales
e : g__Stilbella
f : f__Bionectriaceae
g : g__Clonostachys
h : f__Nectriaceae
i : f__Clavicipitaceae
j : g__Metarhizium
k : o__Coniochaetales
l : f__unclassified_o__Coniochaetales
m : g__unclassified_o__Coniochaetales
n : f__Chaetosphaeriaceae
o : g__Chaetosphaeria
p : f__unclassified_o__Glomerellales
q : g__unclassified_o__Glomerellales
r : f__Microascaceae
s : f__Didymosphaeriaceae
t : g__Paraphaeosphaeria
u : f__Lophiotremataceae
v : g__Lophiotrema
w : c__Leotiomycetes
x : o__Helotiales
y : f__Helotiaceae
z : c__Eurotiomycetes
a1 : o__Chaetothyriales
b1 : f__Herpotrichiellaceae
c1 : f__unclassified_o__Chaetothyriales
d1 : g__unclassified_o__Chaetothyriales
e1 : c__Archaeorhizomycetes
f1 : o__unclassified_c__Archaeorhizomycetes
g1 : f__unclassified_c__Archaeorhizomycetes
h1 : g__unclassified_c__Archaeorhizomycetes
i1 : f__Lipomycetaceae
j1 : g__Lipomyces
k1 : c__unclassified_p__Ascomycota
(b)
LEfSe Bar
CK
LIT
MIT
HIT
p__Rozellomycota
p__Basidiomycota
c__Tremellomycetes
o__unclassified_p__Rozellomycota
f__unclassified_p__Rozellomycota
c__unclassified_p__Rozellomycota
g__unclassified_p__Rozellomycota
f__unclassified_k__Fungi
p__unclassified_k__Fungi
o__unclassified_k__Fungi
o__Tremellales
c__unclassified_k__Fungi
g__unclassified_k__Fungi
f__Trimorphomycetaceae
g__Saitozyma
f__unclassified_o__GS11
o__GS11
g__unclassified_o__GS11
c__Rozellomycotina_cls_Incertae_sedis
o__Hypocreales
o__Helotiales
f__Chaetosphaeriaceae
c__Leotiomycetes
g__Chaetosphaeria
g__Coprinellus
f__Nectriaceae
g__unclassified_o__Platygloeales
c__Eurotiomycetes
g__unclassified_o__Glomerellales
f__Lipomycetaceae
f__Helotiaceae
f__Clavicipitaceae
g__Deconica
f__unclassified_o__Coniochaetales
f__Herpotrichiellaceae
f__unclassified_o__Platygloeales
f__Apiosporaceae
o__Chaetothyriales
g__unclassified_f__Hydnodontaceae
f__Bionectriaceae
g__Metarhizium
f__Lophiotremataceae
g__Clonostachys
f__unclassified_o__Glomerellales
g__Stilbella
g__unclassified_f__Thelephoraceae
g__unclassified_o__Coniochaetales
f__Pyronemataceae
g__Lophiotrema
o__Coniochaetales
c__GS19
g__Lipomyces
g__unclassified_o__GS19
f__Meripilaceae
p__Ascomycota
c__Archaeorhizomycetes
f__unclassified_c__Archaeorhizomycetes
g__unclassified_c__Archaeorhizomycetes
o__unclassified_c__Archaeorhizomycetes
g__unclassified_p__Ascomycota
o__unclassified_p__Ascomycota
f__unclassified_p__Ascomycota
c__unclassified_p__Ascomycota
o__Agaricales
g__unclassified_f__Clavariaceae
f__Clavariaceae
c__Agaricomycetes
f__unclassified_o__Chaetothyriales
g__unclassified_o__Chaetothyriales
f__Didymosphaeriaceae
o__Hymenochaetales
g__Paraphaeosphaeria
f__Microascaceae
0.0 0.5 1.0 1.5 2.0 2.5 3.0 3.5 4.0 4.5 5.0 5.5
LDA SCORE(log10)

Fig. S2 LEfSe analysis of soil fungal abundance in four thinning treatments: LEfSe analysis results of soil fungi (a), and Histogram of LDA scores calculated for the differentially abundant fungi with a threshold value of 3.5 (b).

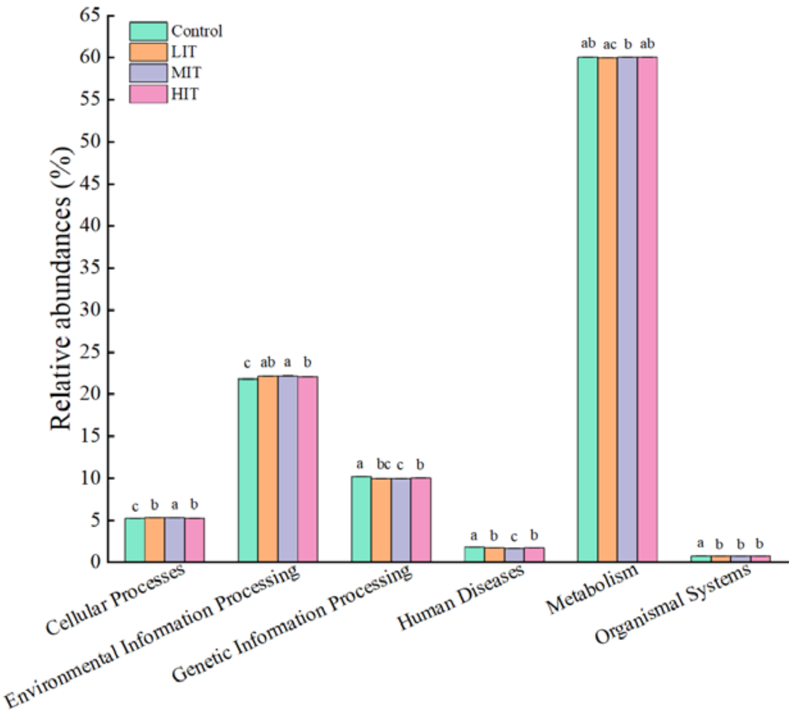

Fig. S3 Variations in composition of bacterial functional groups on OUT inferred by Tax4Fun

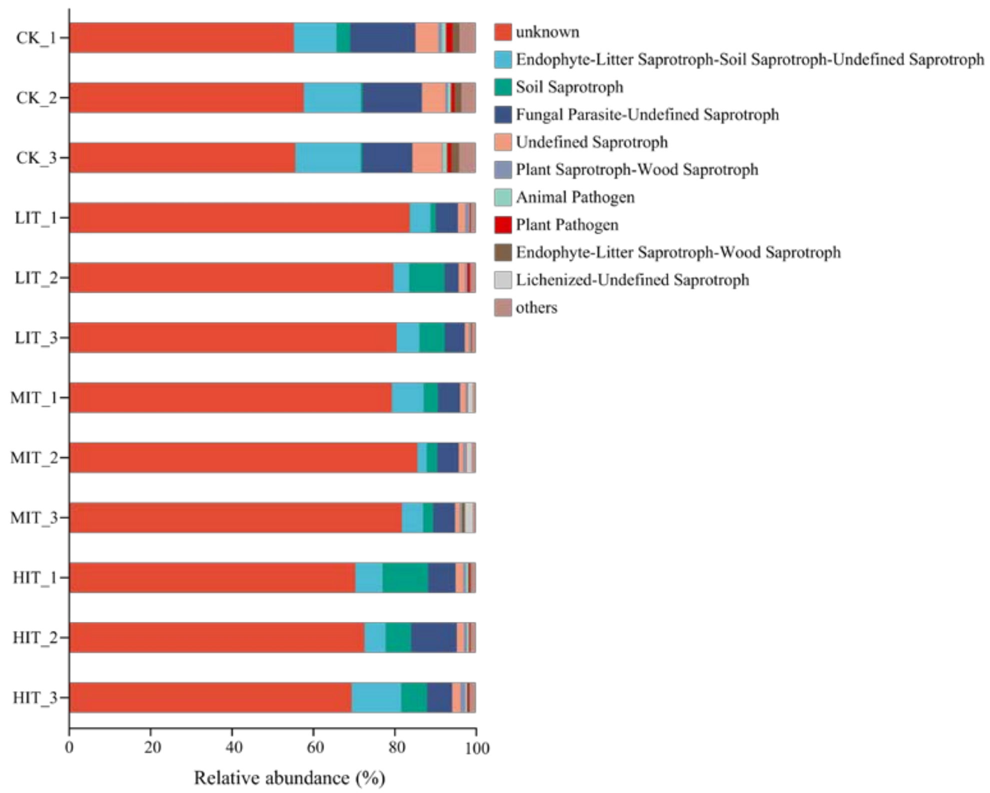

Fig. S4 Variations in composition of fungal functional groups inferred by FUNGuild
